# Bulletin of Medical Science

**Published:** 1847-04

**Authors:** 


### THE BULLETIN OF MEDICAL SCIENCE.

This excellent journal, edited by Dr. Bell, of Philadelphia, has been discontinued. Dr. Bell is one of the most experienced and most gifted of those who have wielded the editorial pen in our country, and his retirement is a loss to our medical literature. We hope his life and energies may long be spared, to be devoted to the service of his profession in other and equally useful departments.

